# Thyroid Hormone Increases TGF-*β*1 in Cardiomyocytes Cultures Independently of Angiotensin II Type 1 and Type 2 Receptors

**DOI:** 10.1155/2010/384890

**Published:** 2010-06-02

**Authors:** Gabriela Placoná Diniz, Marcela Sorelli Carneiro-Ramos, Maria Luiza Morais Barreto-Chaves

**Affiliations:** ^1^Laboratory of Cellular Biology and Functional Anatomy, Department of Anatomy, Institute of Biomedical Sciences, University of São Paulo, 05508-900, São Paulo, Brazil; ^2^Department of Cell and Developmental Biology, Institute of Biomedical Sciences, University of São Paulo, 05508-900, São Paulo, Brazil

## Abstract

TH-induced cardiac hypertrophy *in vivo* is accompanied by increased cardiac Transforming Growth Factor-*β*1 (TGF-*β*1) levels, which is mediated by Angiotensin II type 1 receptors (AT1R) and type 2 receptors (AT2R). However, the possible involvement of this factor in TH-induced cardiac hypertrophy is unknown. In this study we evaluated whether TH is able to modulate TGF-*β*1 in isolated cardiac, as well as the possible contribution of AT1R and AT2R in this response. The cardiac fibroblasts treated with T_3_ did not show alteration on TGF-*β*1 expression. However, cardiomyocytes treated with T_3_ presented an increase in TGF-*β*1 expression, as well as an increase in protein synthesis. The AT1R blockade prevented the T_3_-induced cardiomyocyte hypertrophy, while the AT2R blockage attenuated this response. The T_3_-induced increase on TGF-*β*1 expression in cardiomyocytes was not changed by the use of AT1R and AT2R blockers. These results indicate that Angiotensin II receptors are not implicated in T_3_-induced increase on TGF-*β* expression and suggest that the trophic effects exerted by T_3_ on cardiomyocytes are not dependent on the higher TGF-*β*1 levels, since the AT1R and AT2R blockers were able to attenuate the T_3_-induced cardiomyocyte hypertrophy but were not able to attenuate the increase on TGF-*β*1 levels promoted by T_3_.

## 1. Introduction

The major causes of morbidity and mortality in Western societies have been associated with cardiac hypertrophy and heart failure, which are influenced by several factors [1, 2]. In this context, the cardiomyocytes are known to play important role in the adaptation of myocardial structure and function during cardiac hypertrophy [3–6]. In this sense, several studies have investigated the effects promoted by hypertrophic stimulus in isolated cardiomyocytes in order to evaluate the complex process that coordinates the cardiac hypertrophy. 

Increased thyroid hormone (TH) levels exert important effects on the cardiovascular system, including the induction of cardiac hypertrophy. TH metabolic disarray, as occurs in the hyperthyroidism, has been identified as a high-risk factor for the progression of cardiac diseases and development of heart failure [7]. In addition, TH has been shown to stimulate growth in cardiomyocytes in vitro and in vivo, through a combination of direct and indirect effects, which lead to increased cell size, protein synthesis, and changes in target genes related to cardiac function [8]. In the last decade, some studies demonstrated that the Renin Angiotensin System (RAS) exerts a critical role to the development of the TH-induced cardiac hypertrophy in vivo [9, 10] and in vitro [11]. The Angiotensin II (Ang II), one of the main effectors of the RAS, exerts its effects via the Ang II type 1 (AT1R) and type 2 receptors (AT2R). Although most of hypertrophic effects of Ang II are mediated by the AT1R [12], the AT2R has also been implicated in mediating cardiac growth [13, 14]. 

Another important mediator of the cardiac hypertrophy is the Transforming Growth Factor-*β*1 (TGF-*β*1) [15, 16], which is present in cardiomyocytes and has been implicated in promoting growth of these cells [17]. In this sense, stimulus that triggers the expression of TGF-*β*1 has been associated with an increase on the Ang II levels in the cardiac tissue [18]. Our group recently demonstrated that TH-induced cardiac hypertrophy is accompanied by elevated Ang II levels in vivo and in vitro [11]. Moreover, we also showed that TH-induced cardiac hypertrophy in vivo is accompanied by an increase of cardiac TGF-*β*1 expression, which occurs in dependence on AT1R and AT2R activation [19]. However, the possible contribution of this factor to the TH-induced cardiac hypertrophy is not known. In the same way, the possible involvement of the higher Ang II levels to the TGF-*β*1 modulation induced by T_3_ in isolated cardiac cells, as well as the possible contribution of AT1R and AT2R in this response is also unknown yet. Then, considering that TH and RAS influence several hemodynamic factors and that the heart is composed by several cell types, which could be contributing to the participation of the Ang II receptors in the increase on cardiac TGF-*β*1 levels, the aim of the present paper was to investigate whether TH is able to modulate TGF-*β*1 levels in isolated cardiac cells, as well as the possible contribution of AT1R and AT2R in this process. To address these questions, cardiomyocytes and cardiac fibroblasts cultures were treated with T_3_, simulating a hyperthyroidism condition in vitro. In addition, we used pharmacologic blockers of AT1R and AT2R to evaluate the role of these receptors on TGF-*β*1 levels, as well as in T_3_-induced cardiomyocyte hypertrophy.

Herein, we showed that the cardiomyocytes treated with T_3_ presented higher TGF-*β*1 expression levels. In addition, we confirmed that T_3_ promotes cardiomyocyte hypertrophy in part via AT1R and AT2R. However, the T_3_-induced increase in TGF-*β*1 expression in cardiomyocytes was not changed by the use of AT1R and AT2R blockers. These results indicate, for the first time, that the local Ang II receptors, present on cardiomyocytes, are not implicated in T_3_-induced increase in TGF-*β* expression in these cells. In addition, these data suggest that the trophic effects exerted by T_3_ on cardiomyocytes do not depend on the higher TGF-*β*1 levels, as observed previously for us in vivo, since the AT1R and AT2R blockers were able to attenuate the T_3_-induced cardiomyocyte hypertrophy but were not able to modulate the increase in TGF-*β*1 levels promoted by T_3_ in these cells.

## 2. Material and Methods

### 2.1. Cardiac Myocytes and Fibroblasts Cultures

All protocols were performed in accordance with the Ethical Principles in Animal Research set forth by the Brazilian College of Animal Experimentation and were approved by the Biomedical Sciences Institute/USP Ethics Committee for Animal Research. Primary cultures of neonatal rat ventricular cardiomyocytes were prepared by enzymatic disaggregation, as described previously by us in [20]. To segregate myocytes from nonmyocytes, the dissociated cells were layered into discontinuous Percoll (Amersham Biosciences, Sweden) density gradient. The cells were collected and washed to remove all traces of Percoll. Cellular viability was estimated by Trypan blue method. Cardiomyocytes were cultured in Dulbecco's modified Eagle's medium (DMEM, GIBCO) containing 5% newborn calf serum (NCS; Invitrogen, Carlsbad, CA) and 10% horse serum (Invitrogen). The cardiac fibroblasts were cultured in DMEM containing 10% fetal bovine serum (Invitrogen), which in confluence, were submitted subsequently to subculture. Cells from passage 1 through 2 were grown to confluence and then used in the experiments. The cells were maintained in a humidified incubator (5%  CO_2_, 37°C).

### 2.2. Treatment of the Cells

72  hours after plating, the isolated cardiac cells were maintained in DMEM containing serum 0.5% for 24  hours. Then, the cardiomyocytes and cardiac fibroblasts cultures were treated with serum-free medium containing T_3_ (10 nM, Sigma, St. Louis, MO) or only serum-free medium in control cells for 24  hours. To evaluate the possible role of AT1R and AT2R on cardiomyocytes cultures, the cells were treated with serum-free medium containing T_3_ (10 nM), T_3_ plus Losartan (Los, 1 *μ*M, Sigma Chemical), T_3_ plus PD123319 (PD, 1 *μ*M, Sigma Chemical) or only serum-free medium in control cells for 24  hours.

### 2.3. Protein Synthesis

Protein synthesis on cardiomyocytes cultures was quantified on the basis of tritiated leucine incorporation [21]. Six hours before harvest, L- *[*3,4,5-^3^H*]* leucine (5 *μ*Ci/ml) was added to the culture medium to measure incorporation into newly synthesized protein. Total cellular proteins were precipitated in ice-cold 10% trichloroacetic acid and collected by centrifugation (14000 X g, 10 min, 4°C). The proteins pellets were washed twice by resuspension in cold 10% trichloroacetic acid and collected by centrifugation. The final pellets were dissolved in 0.2 N NaOH by incubation at 60°C for 30 min. The radioactivity was measured by liquid scintillation counting and normalized by the total cell number.

### 2.4. Western Blotting

Protein expression of TGF-*β*1 and *α*-actinin was determined by Western Blotting experiments. Total proteins from cell cultures were obtained using digestion buffer (KCl 90 mM, Hepes 10 mM, MgCl^2+^ 3 mM, EDTA 5 mM, glycerol 1%, DTT 1 mM, SDS 0.04%). Protein concentrations were analyzed by the Bradford method (Bradford 1976). One hundred *μ*g of total protein was resolved by electrophoresis on 5% stacking/10% polyacrylamide-SDS gels, and the resolved proteins were transferred to nitrocellulose membrane (Bio-Rad, CA, USA). The membrane was stained with Ponceau solution to demonstrate that the protein concentration was similar in the different samples. The membrane was then washed with TBST (Tris 50 mM, NaCl 150 mM, pH 7.5 and Tween-20 2%) for 10 minutes at room temperature. After this, the membrane was incubated at 4°C overnight with polyclonal antibody against TGF-*β*1 (Santa Cruz Biotechnology, CA, USA, SC-146, 1 : 500) and *α*-actinin (Santa Cruz Biotechnology, SC-15335, 1 : 1000) in TBST. After washing the membrane, the secondary antirabbit or antigoat I gG antibody conjugated with peroxidase (Amersham Biosciences, New Jersey, USA) at a 1 : 10000 dilution in TBST was added for 1  hour at room temperature. The membrane was washed again with TBST and was incubated with ECL detection reagents (Amersham Biosciences), which produced a chemiluminescence signal that was detected by exposure to X-ray film. The protein bands were quantified by densitometry and the band density was then calculated. The TGF-*β*1 protein expression was normalized by *α*-actinin expression. The values are expressed in percentage in relation to control.

### 2.5. Gene Expression Analysis

Total RNA from cardiomyocytes and fibroblasts cultures were isolated with Trizol Reagent (Invitrogen, CA, USA) in accordance with the manufacturer's instructions. The cDNA species were synthesised with SuperScript II (Life Technologies) from 2 *μ*g of total RNA in a total volume of 20 *μ*L with an oligo (dT) primer. The cDNA reactions were performed for 1  hour at 42°C and stopped by boiling for 5 minutes. The cDNA was used as a template for PCR with specific primers for TGF-*β*1. A 210-bp sequence of the *β*-actin mRNA was chosen as an internal standard RNA for PCRs. To ensure that we were working in conditions of nonsaturation for the PCR reaction, a cycle curve was first made for each gene, since this is a semiquantitative method. Two microlitres of the RT reaction mix were used for PCR in a total volume of 25 *μ*l using the concentration of 0.5 *μ*M of each primer indicated below, together with 50 *μ*M of dNTP and 1 U of Taq polymerase (Life Technologies) in the supplied reaction buffer. The primer of *β*-actin used in the reaction was diluted (1 : 20). The PCR cycling conditions were as follows: for TGF-*β*1: 1 minute at 94°C, 1 minute at 64°C, and 1 minute at 72°C (498-bp amplification product in 21 cycles); and for *β*-actin: 1 minute at 94°C, 1.5 minute at 59.8°C, and 1 minute 30 s at 72°C (210-bp amplification product in 34 cycles). All PCRs were carried out for an initial 3-minutes denaturation step at 94°C and a final 10-minutes extension at 72°C. The following set of primers were used: 5′ AAT ACG TCA GAC ATT CGG GAA GCA 3′ and 5′ GTC AAT GTA CAG CTG CCG TAC ACA 3′ for TGF-*β*1; 5′-TAT GCC AAC ACA GTG CTG TCT GG*- *3′ and 5′-TAC TCC TGC TTC CTG ATC CAC AT*- *3′ for *β*-actin. Oligonucleotides were obtained from Imprint Genetix (São Paulo, Brazil). Ten microlitres of the PCR product were analyzed on a 1.5% agarose gel. Using an Image Analysis System, the densitometric intensities of the TGF-*β* and *β*-actin bands were converted to numeric values. The mRNA expression is expressed as mRNA of the gene of the interest, and the mRNA/*β*-actin ratio is expressed as percentage.

### 2.6. Statistical Analysis

The data obtained are presented as mean ±  SD of at least three independent experiments and are expressed in percentage in relation to control. The number of experiments (*n*) refers to the number of different cell extractions and each experiment was performed in triplicate. Data were analyzed using one-way analysis of variance, followed by Tukey's post hoc test, and values of *P* < .05 were considered statistically significant.

## 3. Results

### 3.1. T_3_ Increases TGF-*β*1 Levels in Cardiomyocytes Cultures

**Figure 1:**
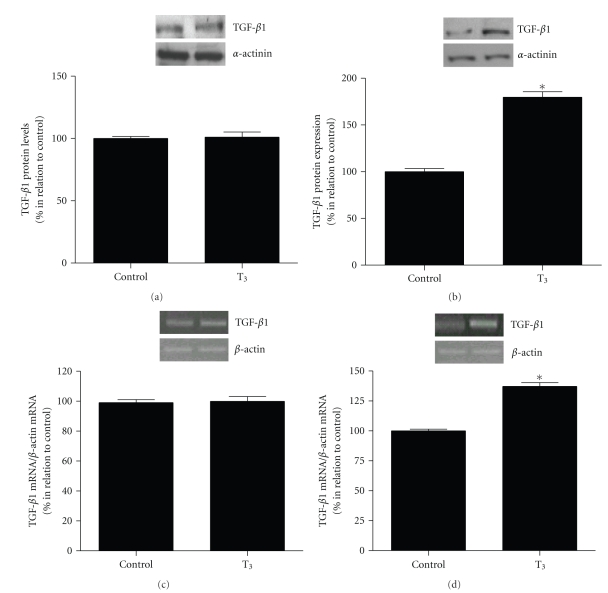
TGF-*β*1 protein expression in cardiac fibroblasts (a) and cardiomyocytes cultures (b), evaluated by Western Blot. TGF-*β*1 gene expression in cardiac fibroblasts (c) and cardiomyocytes cultures (d), evaluated by RT-PCR. The cells were treated for 24  hours with serum-free medium containing 10 nM of T_3_ (T_3_) or only serum-free medium in control cells. *α*-actinin was used for normalization of TGF-*β*1 protein expression and *β*-actin was used for normalization of TGF-*β*1 gene expression. The values are expressed in percentage. * versus control *P* < .01 (*n* = 4).

We have previously demonstrated that TH-induced cardiac hypertrophy was accompanied by elevated cardiac TGF-*β*1 protein expression [19]. In order to evaluate whether TH would be able to modulate directly TGF-*β*1 in isolated cardiac cells, we analyzed its protein and gene expression in cardiomyocytes cultures and in cardiac fibroblasts cultures, using, respectively, western Blotting and RT-PCR analysis (Figure 1). The cardiac fibroblasts submitted to T_3_ (10 nM) treatment for 24  hours did not present alteration on TGF-*β*1 protein and gene expression compared to control cells (Figures 1(a) and 1(c)). However, the cardiomyocytes treated with T_3_ (10 nM) for 24  hours demonstrated a significant increase on TGF-*β*1 protein and gene expression (*P* < .01) in relation to control cells (Figures 1(b) and 1(d)).

These results demonstrate that T_3_ increases the TGF-*β*1 protein and gene expression in cardiomyocytes cultures.

### 3.2. T_3_ Induces Cardiomyocyte Growth via AT1R and AT2R

**Figure 2:**
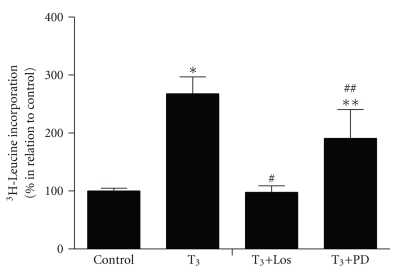
^3^H-Leucine incorporation in cells treated with serum-free medium containing 10 nM of T_3_ (T_3_), as well as in cells treated with 10 nM of T_3_ plus 1 *μ*M of Losartan (Los + T_3_), 10 nM of T_3_ plus 1 *μ*M of PD123319 (PD + T_3_) for 24  hours or only serum-free medium in control cells. Six hours before harvest, L *[*3,4,5-^3^H*]*leucine (5 *μ*Ci/ml) was added to the culture medium to measure incorporation into newly synthesized protein. Results are expressed as percentages. *P < .01 versus control, **P < .05 versus control, #*P* < .01 versus T_3_, ##*P* < .05 versus T_3_ (*n* = 3).

We have previously demonstrated that AT1R acts as key mediator in the TH-mediated cardiac hypertrophy in vivo [9] and in vitro [11]. In addition, the AT2R was also shown to participate in this cardiac hypertrophy model. To confirm the participation of AT1R and AT2R in T_3_-induced cardiomyocyte hypertrophy, we analyzed the protein synthesis as an indicator of hypertrophy (Figure 2). To address these questions, the cells were treated with T_3_ (10 nM), T_3_ plus Losartan (1 *μ*M), an AT1R blocker, or T_3_ plus PD123319 (1 *μ*M), an AT2R blocker. As expected, the cardiomyocytes treated with T_3_ for 24  hours presented a significant increase in protein synthesis (*P* < .01) in relation to control cells. However, protein synthesis was significantly lower in the cardiomyocytes treated with T_3_ plus Losartan (*P* < .01) than in those only treated with T_3_. Similar results were obtained from the cells treated with T_3_ plus PD123319, which demonstrated a significant reduction on protein synthesis (*P* < .05) when compared to T_3_  -treated cells. However, the cardiomyocytes treated with T_3_ plus PD123319 presented a significant increase on protein synthesis (*P* < .05) in relation to control cells.

These data confirm the hypertrophic effect exerted by T_3_ on cardiomyocytes, as well as the important contribution of AT1R and AT2R in this process.

### 3.3. T_3_ Increases TGF-*β*1 in Cardiomyocytes Independent of AT1R and AT2R

**Figure 3:**
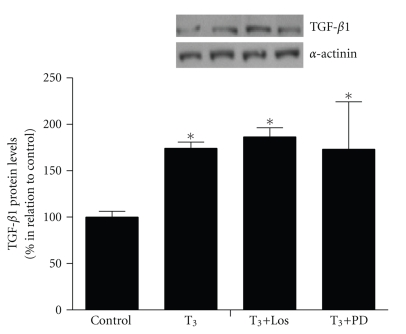
TGF-*β*1 protein expression in cardiomyocytes cultures evaluated by Western Blot in cells treated with serum-free medium containing 10 nM of T_3_ (T_3_), as well as in cells treated with 10 nM of T_3_ plus 1 *μ*M of Losartan (Los + T_3_), 10 nM of T_3_ plus 1 *μ*M of PD123319 (PD + T_3_) for 24  hours or only serum-free medium in control cells. *α*-actinin was used for normalization of TGF-*β*1 expression. The values are expressed in percentage.*versus control *P* < .01 (*n* = 3).

We have previously showed that TH-induced cardiac hypertrophy in vivo is accompanied by increased cardiac TGF-*β*1 levels, which is mediated by AT1R and AT2R [19]. To investigate whether the T_3_-induced increase on TGF-*β*1 expression in cardiomyocytes cultures might be mediated by the local Ang II receptors, present in these cells, as well as the possible participation of the TGF-*β*1 to the trophic effects exerted by T_3_ on cardiomyocytes, we also evaluated the participation of AT1R and AT2R in the T_3_-induced increase on TGF-*β*1 expression, using specific blockers (Figure 3). The cardiomyocytes cultures treated with T_3_ (10 nM) plus Losartan (1 *μ*M) demonstrated a significant increase in TGF-*β*1 expression (*P* < .01) in relation to control values. In the same way, the cardiomyocytes cultures treated with T_3_ (10 nM) plus PD123319 (1 *μ*M) presented higher TGF-*β*1 expression (*P* < .01) compared to control cells. 

These results demonstrate that AT1R and AT2R do not mediate the T_3_-induced increase in TGF-*β*1 protein expression in cardiomyocytes cultures.

## 4. Discussion

Alterations on TH levels are able to influence cardiac function in different routes [22]. In this context, increased TH levels have been shown to promote cardiac hypertrophy [23] and Ang II receptors have a key role in mediating this process [9, 11]. In addition, TH levels have also been shown to influence the cardiac Ang II receptors expression [24]. We have previously described that TH-induced cardiac hypertrophy is accompanied by elevated cardiac TGF-*β*1 levels [19]. However, the exact mechanism by which TH increases the cardiac TGF-*β*1 levels is unknown. Considering this, the present was designed to evaluate whether TH would be able to modulate directly TGF-*β*1 expression in isolated cardiac cells, as well as the possible contribution of AT1R and AT2R to this response. Our results demonstrated that T_3_ induces an increase in TGF-*β*1 protein and gene expression in cardiomyocytes cultures but does not change the TGF-*β*1 protein and gene expression in cardiac fibroblasts cultures.

In order to confirm the critical role exerted by the Ang II receptors to the development of T_3_-induced cardiomyocyte hypertrophy, we evaluated the protein synthesis of cardiomyocytes treated with T_3_ plus AT1R blocker or T_3_ plus AT2R blocker. Herein, we confirmed again that T_3_ stimulates cardiomyocyte hypertrophy via AT1R, since the use of a pharmacologic blocker of this receptor totally prevented the T_3_-induced cardiomyocyte hypertrophy. In addition, our results demonstrated that AT2R also contributes to the development of cardiomyocyte growth promoted by T_3_, since the use of a pharmacologic blocker of this receptor minimized the T_3_-induced hypertrophy. This result is in agreement with some recent studies of the literature, which have implicated the AT2R as a mediator of cardiac growth in vivo and in vitro [13, 14]. However, the participation of AT1R to the T_3_-induced cardiomyocyte growth seems to be more accentuated than the participation of AT2R, suggesting that the AT1R is the main receptor responsible to mediate the trophic effects exerted by Ang II in these cells submitted to T_3_ treatment. This is an important question, which should be investigated. 

We have previously demonstrated that the Ang II receptors mediate the increase in cardiac TGF-*β*1 levels during the TH-induced cardiac hypertrophy in vivo [19]. Considering that TH and RAS influence several hemodynamic factors, which could be responsible for the participation of the Ang II receptors in the TH-induced increase on cardiac TGF-*β*1 levels, in this study we evaluated whether the T_3_-induced increase in TGF-*β*1 expression in cardiomyocytes cultures might be mediated by the local Ang II receptors present in these cells. Herein, we demonstrated that the increase induced by T_3_ on TGF-*β*1 protein expression in cardiomyocytes cultures is not mediated by the Ang II receptors, since the use of AT1R and AT2R blockers did not attenuate the increase on TGF-*β*1 protein expression promoted by T_3_ in these cells. These data suggest that the participation of the Ang II receptors mediating the increase on TGF-*β*1 protein expression in the TH-induced cardiac hypertrophy in vivo probably is not dependent on the local AT1R and AT2R present in the cardiomyocytes. Moreover, considering that the AT1R and AT2R blockers prevented the increase in systolic blood pressure promoted by TH in vivo, as well as the increase in cardiac TGF-*β*1 levels [19], it is plausible that the hemodynamic parameters altered in TH-induced cardiac hypertrophy may be influencing the elevated TGF-*β*1 levels in this model. In addition, several recent studies have emphasized the complexity of cardiac intracellular interactions where heart cells may be both source and target of signals such as cytokines and growth factors [25–27]. In this sense, the preservation of the cardiac architecture in vivo may be critical to the elevated cardiac levels of TGF-*β*1 promoted by TH mediated by Ang II receptors.

Studies using cardiomyocytes cultures indicate that TGF-*β*1 stimulates protein synthesis [28]. In addition, the causal link between Ang II and TGF-*β*1 in cardiac hypertrophy was recently demonstrated [29], since TGF-*β*1 expression is increased by Ang II in cardiomyocyte [30]. In this sense, the myocardial induction of TGF-*β*1 has been focus of particular interest in cardiac biology because this cytokine plays a crucial role in the transition from compensated to de-compensated hypertrophy [31]. Taking this into account, it would be possible to suppose that part of the hypertrophic effect exerted by T_3_ on cardiomyocytes might be mediated by the higher TGF-*β*1 protein expression. However, our data suggest that the trophic effects exerted by T_3_ on cardiomyocytes are not dependent of the higher TGF-*β*1 levels, since the AT1R and AT2R blockers were able to attenuate the T_3_-induced cardiomyocyte hypertrophy but were not able to attenuate the increase on TGF-*β*1 levels promoted by T_3_ in these cells. However, considering that the effects of TGF-*β*1 are mediated by TGFR-*β*1 and TGFR-*β*2 receptors, which are present in cardiomyocytes [32], the existence of the possible action of TH on TGF-*β*1 receptors have to be considered. In this sense, it would be important to evaluate the TH effect on TGF-*β*1 receptors in isolated cardiomyocytes. However, future studies will help in the elucidation of the exact role exerted by the elevated TGF-*β*1 expression in T_3_-treated cardiomyocytes.

## 5. Conclusions

In summary, the data obtained in the present study demonstrate for the first time that T_3_ increases TGF-*β*1 protein and gene expression in cardiomyocytes cultures. In addition, the results evidenced that the TH-induced increase on TGF-*β*1 levels in cardiomyocytes cultures is not dependent on local AT1R and AT2R present in these cells. Finally, these data suggest that the trophic effects exerted by T_3_ on cardiomyocytes are not dependent on the higher TGF-*β*1 levels, since the AT1R and AT2R blockers were able to attenuate the T_3_-induced cardiomyocyte hypertrophy but were not able to attenuate the increase on TGF-*β*1 levels promoted by T_3_ in these cells.
